# 3-Hydroxyacyl CoA Dehydratase 2 Is Essential for Embryonic Development and Hepatic Metabolic Function Under a Low-Fat, High-Carbohydrate Diet

**DOI:** 10.3390/biology14060712

**Published:** 2025-06-17

**Authors:** Lengyun Wei, Fengli Wang, Luoxue Hua, Qun Wang, Benfei Hu, Ziye Yang, Letao Li, Chenfeng Liu, Kezhen Wang

**Affiliations:** 1 School of Life Science, Anhui Medical University, Hefei 230032, China; weilengyun@ahmu.edu.cn (L.W.); 2245010270@stu.ahmu.edu.cn (F.W.); hualouxue@126.com (L.H.); 2445010284@stu.ahmu.edu.cn (Q.W.); 2213180008@stu.ahmu.edu.cn (B.H.); ahmuziye@163.com (Z.Y.); llt13905627539@163.com (L.L.); 2 Wuxi School of Medicine, Jiangnan University, Wuxi 214122, China

**Keywords:** Hacd2, embryonic development, LFHCD, hepatic dysfunction, adipose tissue formation

## Abstract

The conversion of carbohydrates into fatty acids is central for energy storage and the development and functioning of organs. Our previous study revealed that *Hacd2* deficiency alleviates the fatty liver and diabetes induced by HFD. This study aimed to explore the roles of Hacd2 in organ development and metabolic homeostasis under an LFHCD, which still need to be more deeply explored. We found that the germline deletion of *Hacd2* impairs long-chain fatty acid synthesis, which caused embryonic abnormalities after 7.5 days and led to embryonic lethality, as confirmed via photograph and hematoxylin-eosin staining. We next constructed *Hacd2LKO* mice and found that *Hacd2LKO* mice were largely normal when fed a chow diet, except for reduced inguinal white adipose tissue formation and glucose metabolism. Meanwhile, under an LFHCD, *Hacd2* deletion markedly controlled body weight and white adipose tissue formation, leading to lower cholesterol and triglycerides in serum; however, it unexpectedly resulted in enlarged liver volume, hepatocyte swelling and nuclear abnormalities, and infiltration of inflammatory cells, including macrophages, neutrophils and dendritic cells. Furthermore, inhibition of Hacd2 also reduced triglyceride levels and the expression of related lipogenic genes during adipocyte differentiation, as confirmed via RNA interference analysis. These findings highlight the critical roles of Hacd2 in embryonic development and metabolic diseases, revealing its protective function in maintaining liver homeostasis under an LFHCD. Therefore, targeted interventions involving Hacd2 for metabolic diseases must take into account dietary changes and the functioning of the liver.

## Simple Summary

This study investigates the roles of 3-hydroxyacyl CoA dehydratase 2 (Hacd2)—a key enzyme in fatty acid elongation—in embryonic development and metabolic homeostasis. Our previous research indicated that liver-specific *Hacd2* ablation (*Hacd2LKO*) protects mice against high-fat diet (HFD)-induced obesity, hepatic steatosis and diabetes. Using germline *Hacd2*-knockout mice, we now establish that Hacd2 is essential for embryonic development. Moreover, through *Hacd2LKO* studies, we reveal that Hacd2 plays a protective role in maintaining hepatic homeostasis under low-fat, high-carbohydrate diet (LFHCD) conditions. Together, these findings highlight the importance of considering diet-specific hepatic dysfunctions when targeting Hacd2 for therapeutic interventions.

## 1. Introduction

An organism’s metabolism balances the conversion of nutrients, such as that from glucose to fatty acids and triglycerides, under an excessive intake of carbohydrates, which are finally transformed into fats and stored as adipose tissue. Fats serve dual roles: as energy reserves (e.g., for thermogenesis via adipose tissue) and as endocrine regulators, secreting hormones such as leptin while supporting embryonic development through adipose-derived stem cells. However, the excessive accumulation of fat triggers obesity and metabolic disorders, underscoring the need to study fatty acid synthesis for the prevention of such diseases. Fatty acid biosynthesis in mammals initiates with the production of saturated fatty acids (2C-16C) by soluble fatty acid synthase (FASN), followed by the elongation of 16C fatty acids into very long-chain fatty acids (VLCFAs) via endoplasmic reticulum (ER)- and mitochondrial-localized fatty acyl-CoA elongases [1]. In particular, Hacd2 serves as a rate-limiting enzyme in the ER-based very long-chain fatty acid (VLCFA) elongation pathway, specifically mediating the third enzymatic step required for the synthesis of VLCFAs such as palmitoleic acid and eicosapentaenoic acid (EPA) [2]. A recent study in *Hacd2LKO* mice has revealed ameliorated obesity, hepatic steatosis, and diabetes in HFD-treated mice, which were achieved by upregulating thermogenic genes to enhance energy expenditure, positioning Hacd2 as a therapeutic target [3]. Beyond metabolism, Hacd2 non-enzymatically binds PRKN (Parkin), stabilizing PKM2 to activate c-Myc-driven glycolysis, thereby activating c-Myc-driven glycolysis to promote tumor cell proliferation [4]. However, the role of Hacd2 and the associated signal pathways are still not fully understood.

The liver is a vital metabolic organ in the body, playing critical roles in biosynthesis and detoxification. It synthesizes essential metabolism-related enzymes and bile acids while dynamically balancing the metabolism of nutrients, including carbohydrate to lipid conversion and energy storage, under diverse dietary conditions. However, chronic metabolic load and stress can lead to liver dysfunction or damage; for example, prolonged HFD consumption induces pathological alterations, including hepatocyte ballooning degeneration and lipid droplet accumulation, clinically defined as metabolic dysfunction-associated fatty liver disease (MAFLD). MAFLD exacerbates insulin resistance and hepatic gluconeogenesis, thus causing hyperglycemia while triggering inflammation and fibrosis through Kupffer cell activation, hepatic stellate cell proliferation and collagen deposition, which lead to cirrhosis [5,6]. Beyond MAFLD induced by an HFD, whether a simple high-carbohydrate diet can induce liver dysfunction and which molecules are involved in protecting the liver from damage under LFHCD conditions still require detailed exploration.

In this study, to identify the roles of Hacd2 in controlling embryo development and maintaining metabolic homeostasis and liver function under an LFHCD, we constructed mice with systemic or liver *Hacd2* knockout to investigate morphological, metabolic, adipose tissue formation-related and inflammatory alterations. Our study demonstrates the indispensable role of Hacd2 in regulating metabolic function under an LFHCD.

## 2. Materials and Methods

### 2.1. Generation of Hacd2^−/−^ and Hacd2LKO Mice and Housing Conditions

*Hacd2*^+/−^ mice were obtained from Shanghai Model Organisms Center, Inc. (Shanghai, China), while *Hacd2^flox/flox^* mice were generated by Cyagen Biosciences (Suzhou, China). To produce *Hacd2*^−/−^ embryos, we intercrossed *Hacd2*^+/−^ males and females. *Hacd2LKO* mice were generated by crossing *Hacd2*^*flox/flox*^ mice with Alb-Cre mice (C57BL/6J background; Jackson Laboratory, stock #003574), as previously described [3]. All mice were maintained under standard housing conditions in a temperature-controlled facility with a 12 h light-dark cycle. Animals had ad libitum access to water and were fed either standard chow or an LFHCD (Trophic Animal Feed High-Tech Co., Ltd., Nantong, China). For tissue collection, mice were euthanized by isoflurane overdose. Tissues and serum were promptly harvested, flash-frozen in liquid nitrogen, and stored at −80 °C. All animal procedures were approved by the Institutional Animal Care and Use Committee of Jiangnan University, Ethics No. (Ethics Approval No. JN. No 20180615c0041230; approval date: 5 June 2018).

Genotyping for Cre transgenes was performed by PCR using tail-derived genomic DNA with the following primers: forward 5′-ATTTGCCTGCATTACCGGTCG-3′ and reverse 5′-CAGCATTGCTGTTCACTTGGTC-3′. The PCR protocol consisted of an initial denaturation at 94 °C for 2 min, followed by 35 cycles of 94 °C for 30 s, 58 °C for 30 s, and 72 °C for 30 s, with a final extension at 72 °C for 10 min.

*Hacd2* genotyping was conducted using a three-primer system: F1 5′-CCTGTCCCACTGAGGCACTTGTA-3′, F2 5′-GCAGGAACGGTGAGCATATTTGG-3′, and R 5′-GTTGGATTTCTCTCAGCTGCACTT-3′. The PCR conditions included initial denaturation at 94 °C for 3 min, 33 cycles of 94 °C for 30 s, 62 °C for 35 s, and 72 °C for 35 s, followed by final extension at 72 °C for 5 min.

*Hacd2^flox/flox^* genotyping was performed by PCR (Initial denaturation at 94 °C for 5 min, 35 cycles at 94 °C for 30 s, 60 °C for 35 s, and 72 °C for 1 min, and final extension at 72 °C for 5 min), with primers: P1 5′-GAGCCGAGTTCTAGACAAGTGAG-3′; P2 5′-AAGACCCGAGATTTACCAAGTG-3′; P3 5′-TTTGCATTGGCCTTGTTCTCAG-3′ and P4 5′-TCAGGCAGGTTCAGCTCAATCTC-3′.

### 2.2. Experimental Mouse Design

All mouse experiments in this study adhered to the 3R principle (Replacement, Reduction, and Refinement). For embryonic development analysis, we crossed *Hacd2*^+/−^ females with *Hacd2*^+/−^ males. Embryos were collected from 28 pregnant mice at indicated time points for morphological analysis and liver histology (H&E) and from an additional 12 pregnant mice for fatty acid analysis. For dietary experiments, mice were weaned at 4 weeks of age and maintained on a standard chow diet for 6 weeks until they reached approximately 10 weeks of age (~25 g body weight) before metabolic testing. The normal chow diet, 5 WT and 5 *Hacd2LKO* mice were used to access body size, tissue morphology, and the expression of *Hacd2* across tissues. An additional 4 WT and 4 *Hacd2LKO* mice underwent metabolic tests, including carbohydrate/fat oxidation, energy expenditure, respiratory exchange ratio (RER), insulin tolerance test (ITT) and glucose tolerance test (GTT). In the subsequent LFHCD diet, 5 WT and 5 *Hacd2LKO* mice were monitored for food intake, body weight, and liver/fat pad mass. H&E, the level of triacylglycerol (TG), total cholesterol (TC), and alkaline phosphatase (ALP) levels were analyzed, along with GTT. In addition, immune cell subsets in the liver are analyzed using 3 WT and 5 *Hacd2LKO* mice under an LFHCD. Mice were randomly assigned by computer-generated allocation. In total, this study analyzed 96 mice, while several mice were used for independent experiments for key conclusions.

### 2.3. Embryo Acquisition and Analysis

*Hacd2*^+/−^ male and female mice were mated, with gestational age determined via vaginal plug observation; embryos at E7.5, E8.5, E9.5, E10.5, E11.5, and E12.5 days post-coitum were dissected from the uterus, their morphological features and sizes observed and photographed using a stereomicroscope, then numbered, with yolk sacs separated to confirm genotypes; some embryos were stored at −80 °C for subsequent fatty acid profile analysis based on identified genotypes, while others were processed for H&E staining.

### 2.4. Low Fat and High Carbohydrate Diet and Feeding

Male mice were maintained on a standard chow diet for six weeks prior to dietary intervention. Subsequently, they were transitioned to either a standard chow diet or an LFHCD produced by Trophc Animal Feed High-Tech Co., Ltd. (Nantong, China). The LFHCD comprised 10% fat and 70% carbohydrates (glucose and fructose) and maintained 20% protein, with nutrient composition validated as previously described [3]. Both diets were pelleted and provided ad libitum via hanging hopper systems for five weeks to facilitate accurate intake monitoring and metabolic assessment.

### 2.5. Histological Analysis

Fresh liver tissues from age-matched WT and knockout mice were fixed in 4% paraformaldehyde (PFA) in phosphate-buffered saline (PBS). The tissues underwent gradual dehydration through an ethanol concentration gradient, followed by clearing in a xylene/absolute alcohol mixture. Samples were then embedded in paraffin after wax immersion. It is then placed in a microtome for sectioning, typically with a thickness of 4–10 microns. Hematoxylin and eosin staining were then performed, and all H&E slices were scanned with a tissue in situ cell scanning analysis system and analyzed with SlideViewer software 4.0.0.

### 2.6. Fatty Acid Methyl Ester (FAME) Analysis

*Hacd2*^+/−^ female and *Hacd2*^+/−^ male mice were bred, while mated females were euthanized at indicated embryonic ages. Embryos were carefully separated using scissors and forceps under a dissecting microscope. The resulting embryos included genotypes WT, *Hacd2*^+/−^, and *Hacd2*^−/−^, which were grouped by genotype confirmation. For each genotype group, seven embryos were collected and homogenized using a tissue grinder to isolate and quantify proteins; total lipids were then extracted via the chloroform-methanol method, followed by GC-MS analysis (Rtx-Wax column: 30 m × 0.25 mm × 0.25 μm; helium carrier gas; 240 °C inlet temperature; temperature program: 150 °C initial hold for 2 min, 5 °C/min ramp to 190 °C for 5 min, then 10 °C/min increase to 220 °C for 16 min; mass range: 50–550 *m*/*z*) for qualitative and quantitative analysis of different fatty acid contents [7,8].

### 2.7. Quantitative Real-Time PCR (qPCR) Analysis

Total RNA was extracted from tissues using the Ultrapure RNA Kit (ComWin Biotech, Jiangsu, China) and reverse transcribed (1 μg RNA input) with the High Capacity cDNA Reverse Transcription Kit (Takara, Shiga, Japan). cDNA was diluted to 100 ng/μL and amplified in 20 μL reactions containing SsoAdvanced™ SYBR^®^ Green Supermix (Bio-Rad, England, UK). Reactions were performed in triplicate on a [specify instrument model, e.g., CFX96 Touch™ Real-Time PCR System]. Gene expression was quantified using the 2^−ΔΔCT^ method with β-actin as the reference gene.

### 2.8. Metabolic Phenotyping by Indirect Calorimetry

10-week-old WT mice and *Hacd2LKO* mice were fed on a normal chow diet and LFHCD for 5 weeks. 15-week-old mice were individually housed in metabolic chambers within a Comprehensive Laboratory Animal Monitoring System (CLAMS; Columbus Instruments) under precise thermostatic control. Following a 24 h acclimation period, we measured oxygen consumption (VO_2_), carbon dioxide production (VCO_2_), and respiratory exchange ratio (RER), with all values normalized to total body weight. We calculated substrate utilization and energy expenditure using established equations: Carbohydrate oxidation: (4.585 × VCO_2_ − 3.226 × VO_2_) × 4 (The multiplier 4 converts mass/time to kcal/time) [9]; similarly, Fat oxidation: (1.695 × VO_2_ − 1.701 × VCO_2_) × 9; Energy expenditure: (3.815 + 1.232 × RER) × VO_2_ (Based on calorific value of oxygen) [10]. Physical activity was continuously monitored using infrared beam-break sensors.

### 2.9. Metabolic Measurements

ITT and GTT were performed in mice after a 2 h (ITT) or 6 h (GTT) fast. Mice received an intraperitoneal bolus injection of either insulin (0.75 U/kg; ITT, MedChemExpress, Monmouth Junction, NJ, USA) or glucose (1.5 g/kg; GTT, MedChemExpress, Monmouth Junction, NJ, USA). Blood glucose levels were measured from tail vein samples at specified time points using a handheld glucometer (Abbott, Chicago, IL, USA).

### 2.10. Biochemical Analyses

Serum and hepatic triacylglycerol (TG), total cholesterol (TC) and alkaline phosphatase (ALP) activity were quantified using commercial assay kits according to its instructions (TG, TC and ALP: Nanjing Jiancheng Bioengineering Institute, Nanjing, China).

### 2.11. Flow Cytometry

After the mice were sacrificed, the liver was removed, minced with scissors and transferred to a 15 mL centrifuge tube, where it was digested simultaneously at 37 °C for 60 min with 1 mg/mL collagenase Type 4 (Worthington Biochemical, Lakewood, NJ, USA) and 0.1 mg/mL DNase I (Sangon Biotech, Shanghai, China). Subsequently, the liver tissue is triturated and centrifuged to obtain a pellet. After resuspension by adding 42% percoll (Cytiva, Uppsala, Sweden) to the pellet, add 70% percoll. The cells in the buffy coat at the junction are then taken, which are called immune cells. Cells were stained at 4 °C for 15 min with flow cytometry antibodies (CD45 (B220) Monoclonal Antibody (RA3-6B2), ebioscience, 45-0452-82; anti-mouse/human CD11b Antibody, Biolegend, 101212; anti-mouse CD11c Antibody, Biolegend, 117310; F4/80 Monoclonal Antibody (BM8), ThermoFisher, 47-4801-82; Ly-6G/Ly-6C Monoclonal Antibody (RB6-8C5), eBioscience, RB6-8C5; MHC Class II (I-A/I-E) Monoclonal Antibody (M5/114.15.2), eBioscience, 47-5321-82). Flow cytometry data collection was followed by LSR Fortessa (BD Biosciences, Franklin Lakes, NJ, USA).

### 2.12. Statistical Analysis

All the biological replicates (n = 3–7) in this study were included per group for analyses comparing WT and KO or *Hacd2LKO* groups. Statistical values were statistically processed using GraphPad Prism software 8.0. For single comparisons between two groups, significance was assessed using an unpaired two-tailed Student’s t-test. H&E staining results were graphically processed using Case Viewer software 3.0. Data are presented as mean ± SEM, and statistical significance was set at *p* < 0.05, with significance levels denoted by asterisks in Figures (* *p* < 0.05, ** *p* < 0.01, *** *p* < 0.001, **** *p* < 0.0001). ns indicates no significant differences, and the data were organized and summarized using Adobe Illustrator software v27 (2023).

## 3. Results

### 3.1. Germline Deletion of Hacd2 Causes Embryonic Lethality

**Figure 1:**
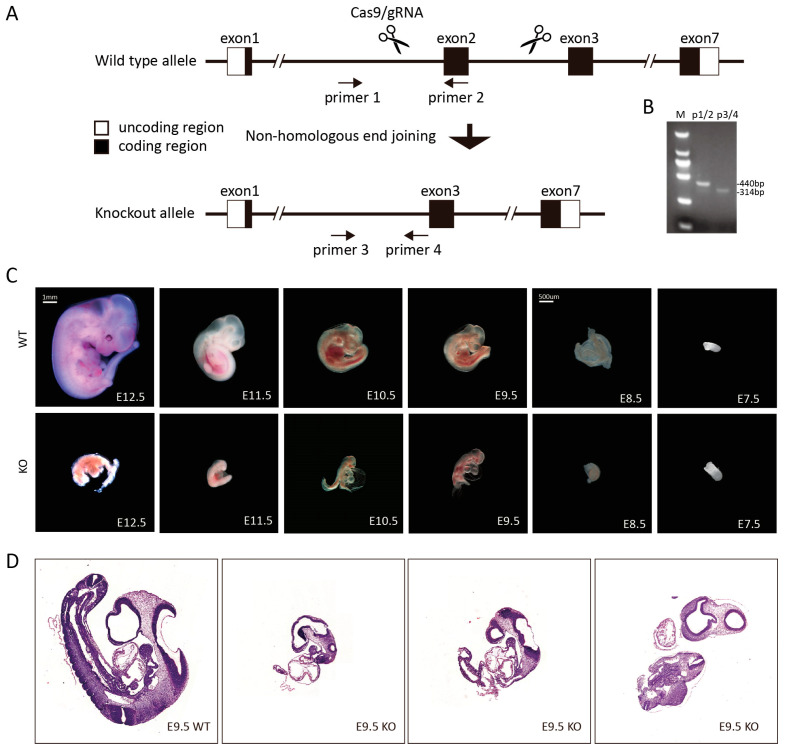
*Hacd2* deficiency causes embryonic lethality. (**A**,**B**) *Hacd2* was deleted via Cas9-mediated gene targeting. (**C**) *Hacd2* deficiency in mice led to embryonic lethality. (**D**) H&E-stained sections of embryonic day 9.5 (E9.5) WT and *Hacd2*^−/−^ mice.

To explore the roles of Hacd2 in metabolic and physiological homeostasis, we generated *Hacd2*-deficient mice using CRISPR/Cas9 technology by microinjecting single-guide RNAs (sgRNAs) targeting *Hacd2* and Cas9 mRNA into the cytoplasm of mouse zygotes (Figure 1A). PCR genotyping identified F0 founder mice carrying frame-shifted mutations in the *Hacd2* gene, which were then bred with C57BL/6J mice to establish heterozygous F1 offspring (*Hacd2*^+/−^) (Figure 1B). To obtain homozygous knockout mice, we performed three types of crosses: wild-type males × *Hacd2*^+/−^ females, *Hacd2*^+/−^ males × wild-type females and *Hacd2*^+/−^ intercrosses (Table 1). Genotypic analysis showed Mendelian segregation in the first two crosses, with 48.6% (68/140) *Hacd2*^+/+^ and 51.4% (72/140) *Hacd2*^+/−^ in wild-type × heterozygous crosses; and 50.7% (68/134) *Hacd2*^+/+^ and 49.3% (66/134) *Hacd2*^+/−^ in the reciprocal cross. However, in *Hacd2*^+/−^ intercrosses, the genotype distribution was 38.3% (169/441) *Hacd2*^+/+^ and 61.7% (272/441) *Hacd2*^+/−^, with no *Hacd2*^−/−^ offspring detected (Table 1). This deviation from the expected Mendelian 1:2:1 ratio suggests that homozygous *Hacd2* deletion results in embryonic lethality.

To investigate the timing of embryonic lethality in *Hacd2*^−/−^ mice, we performed timed dissections of pregnant females carrying *Hacd2*^−/−^ embryos along with wild-type (WT) controls. Embryos were collected at embryonic days E7.5 through E12.5 for morphological evaluation. By E12.5, WT embryos displayed normal development, characterized by well-defined ocular structures, skeletal formation and vascular networks. In contrast, *Hacd2*^−/−^ embryos were markedly smaller and exhibited severe developmental abnormalities, indicating that lethality had occurred prior to this stage. Genotyping confirmed the *Hacd2*^−/−^ status of abnormal embryos, and statistical analysis revealed a 3:1 ratio of morphologically normal to abnormal embryos, consistent with Mendelian inheritance (1:2:1), suggesting the selective loss of homozygous mutants. Similar developmental defects were observed at E11.5, E10.5, E9.5 and E8.5, with *Hacd2*^−/−^ embryos showing significant growth retardation and morphological anomalies compared to WT littermates (Figure 1C). Hematoxylin and eosin (H&E) staining at E9.5 further confirmed these findings: WT embryos displayed typical developmental features, including a U-shaped trunk curvature, intact prosencephalic and rhombencephalic ventricles, and normal pericardial morphology. In contrast, *Hacd2*^−/−^ embryos were markedly smaller, with severe truncal malformations, diminished curvature and abnormal forebrain, hindbrain, and pericardial structures. Notably, at E7.5, embryos of both genotypes appeared morphologically comparable in terms of size and developmental stage (Figure 1D). Together, these results demonstrate that *Hacd2*^−/−^ embryos undergo developmental arrest at around E7.5, confirming that homozygous *Hacd2* deletion leads to embryonic lethality.

### 3.2. Hacd2 Knockout Impairs Elongation of C18 to C20 Fatty Acids in Mice

**Figure 2:**
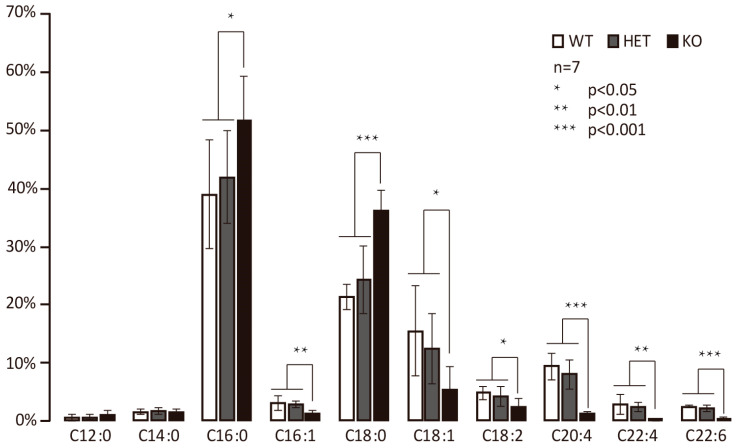
*Hacd2* knockout impairs elongation of C18 to C20 fatty acids in mice.

To investigate the role of Hacd2 in long-chain fatty acid (LCFA) synthesis, we analyzed fatty acid methyl esters (FAMEs) via GC-MS in WT, *Hacd2*^+/−^, and *Hacd2*^−/−^ embryos. No significant differences were observed in C12:0 and C14:0 saturated fatty acid (SFA) levels across genotypes. However, *Hacd2*^−/−^ embryos exhibited significantly increased levels of C16:0 and C18:0 SFAs compared to WT and *Hacd2*^+/−^ littermates (Figure 2). Conversely, levels of unsaturated fatty acids (UFAs) with ≥18 carbon atoms were markedly reduced in *Hacd2*^−/−^ embryos. These findings suggest that *Hacd2* ablation impairs the elongation of C18 fatty acids into C20 species, implicating Hacd2 as a critical enzyme in the synthesis of long-chain fatty acids.

### 3.3. Liver-Specific Hacd2 Deficiency Affects iWAT Formation and Glucose Under Chow Diet

**Figure 3:**
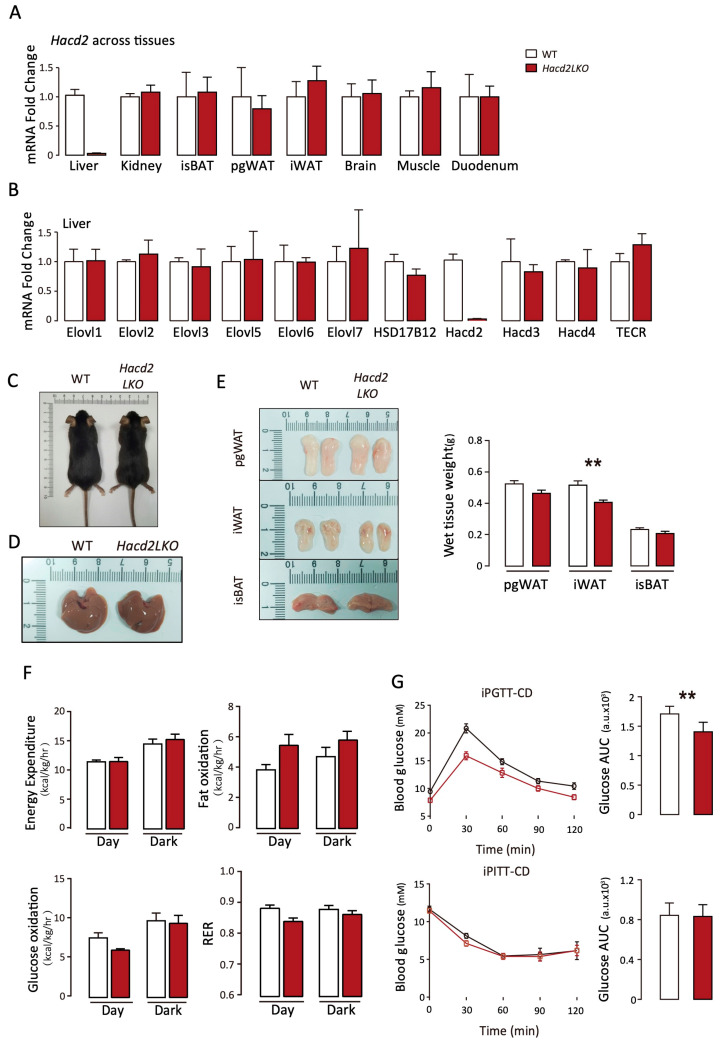
Liver-specific *Hacd2* deficiency affects iWAT formation and glucose under the chow diet. (**A**) Tissue distribution of *Hacd2* mRNA (n = 5). (**B**) Hepatic mRNA expression of fatty acid elongases in WT and *Hacd2LKO* mice (n = 5). (**C**) Representative image of chow-fed WT and *Hacd2LKO* mice. (**D**) Image of livers from chow-fed WT and *Hacd2LKO* mice. (**E**) Adipose tissue weights in WT and *Hacd2LKO* males fed a normal chow diet (n = 5). (**F**) Energy expenditure, fat oxidation rates (kcal/kg/hr; n = 4/group), glucose oxidation rates (kcal/kg/hr; n = 4/group) and respiratory exchange ratio (RER) in WT and *Hacd2LKO* mice during dark/light cycles. (**G**) Intraperitoneal glucose tolerance test (ipGTT) (n = 4) and intraperitoneal insulin tolerance test (ipITT) results with AUC analysis (n = 4). Data are presented as mean ± SEM, and statistical significance: **, *p* < 0.01.

Given the embryonic lethality of *Hacd2*^−/−^ mice and the central role of the liver in metabolic regulation, we sought to investigate the physiological functions of Hacd2-mediated long-chain fatty acid synthesis in the liver. To this end, we generated liver-specific *Hacd2* knockout (*Hacd2LKO*) mice by crossing mice harboring loxP-flanked *Hacd2* alleles with Albumin-cre mice, which express Cre recombinase specifically in hepatocytes [11]. Deletion of *Hacd2* in the liver was confirmed at the mRNA level, with no detectable reduction in *Hacd2* expression in other tissues such as the kidney, adipose tissue, or muscle. Furthermore, *Hacd2LKO* mice did not present altered expression of other key genes involved in lipid metabolism (Figure 3A,B). Under standard chow diet conditions, age-matched male *Hacd2LKO* and WT mice displayed similar body weights, liver morphology and liver mass (Figure 3C,D), along with comparable weights of interscapular brown adipose tissue (isBAT) and perigonadal white adipose tissue (pgWAT). However, *Hacd2LKO* mice exhibited a significant reduction in inguinal white adipose tissue (iWAT) mass (Figure 3E). To assess potential metabolic alterations, we measured whole-body energy expenditure, fatty acid and glucose oxidation, and the respiratory exchange ratio (RER) across light/dark cycles. No significant differences were observed between *Hacd2LKO* and WT mice (Figure 3F). In glucose tolerance tests (GTTs), *Hacd2LKO* mice displayed impaired glucose homeostasis compared to WT controls, while insulin tolerance tests (ITTs) revealed no significant differences in insulin sensitivity (Figure 3G). Collectively, these results suggest that, in *Hacd2LKO* mice, modest metabolic alterations occur under a normal chow diet, characterized by reduced iWAT formation and impaired glucose tolerance, while their overall energy metabolism remains largely unaffected.

### 3.4. Liver-Specific Hacd2 Deficiency Impairs Hepatic Metabolic Homeostasis and Function Under an LFHCD

**Figure 4:**
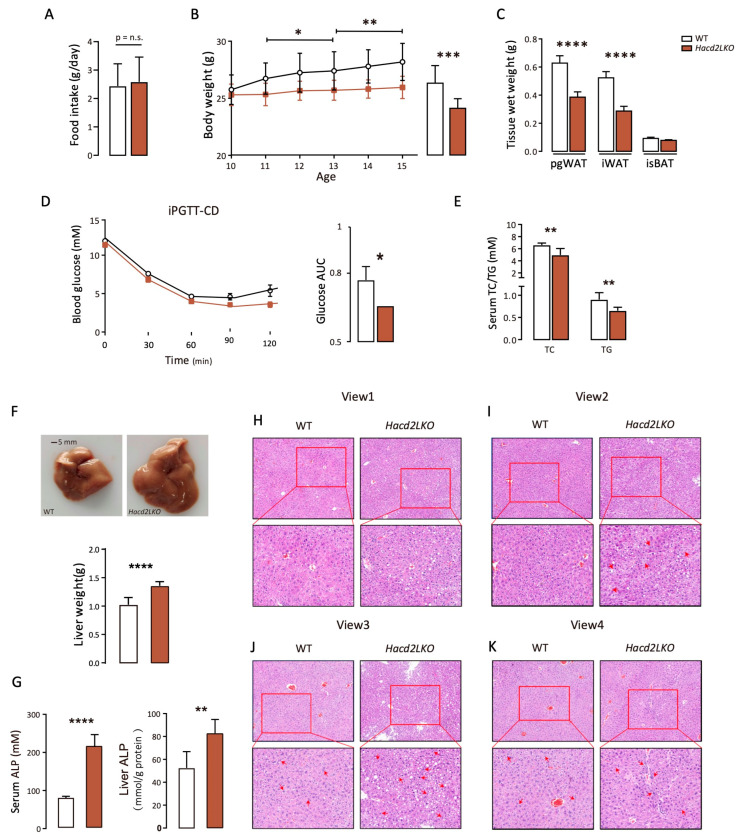
Liver-specific *Hacd2* deficiency impairs hepatic metabolic homeostasis and function under an LFHCD. (**A**) Daily food intake in WT vs. *Hacd2LKO* mice (n = 5). (**B**) Body weight of WT and *Hacd2LKO* mice (n = 5). (**C**) Adipose tissue weights in WT and *Hacd2LKO* mice (n = 5). (**D**) Intraperitoneal glucose tolerance test (ipGTT) results with AUC analysis (n = 5). (**E**) Serum hepatic triglyceride (TG) and total cholesterol (TC) levels in WT and *Hacd2LKO* mice (n = 5). (**F**) Image of liver and liver weight in LFHCD-fed WT and *Hacd2LKO* mice. (**G**) Alkaline phosphatase level in serum and liver (n = 5). (**H**–**K**) Representative images of H&E-stained liver (View 1: hepatic cord; View 2: hepatocyte swelling; View 3: nuclear abnormalities; and View 4: inflammation) and the ruler is in the lower-left corner of the picture (n = 5). Data are presented as mean ± SEM, and statistical significance: *, *p* < 0.05; **, *p* < 0.01; ***, *p* < 0.001, **** *p* < 0.0001. n.s., no significance.

To further investigate the roles of Hacd2 under an LFHCD, we conducted 5-week LFHCD feeding in WT and *Hacd2LKO* mice. Despite comparable daily food intake (Figure 4A), *Hacd2LKO* mice nearly fully controlled their increase in body weight compared to WT mice (Figure 4B). Additionally, *Hacd2LKO* mice exhibited marked reductions in the weight of perigonadal white adipose tissue (pgWAT) and inguinal white adipose tissue (iWAT), underscoring the critical role of Hacd2 in lipid accumulation under carbohydrate-rich conditions (Figure 4C). Serological analyses further revealed decreased blood glucose, as well as lower levels of total cholesterol and triglycerides in *Hacd2LKO* mice relative to WT mice (Figure 4D,E), indicating that *Hacd2* deficiency attenuates metabolic burden under LFHCD stress.

Strikingly, *Hacd2LKO* mice displayed substantial increases in liver volume and mass. Biochemical analyses of serum and liver tissues revealed elevated levels of alkaline phosphatase (ALP) in *Hacd2LKO* mice compared to WT controls (Figure 4F,G). Histopathological examinations further revealed pronounced structural disorganization of the liver, as shown in Figure 4H–K. Notable abnormalities included disrupted hepatic lobule architecture, disordered hepatic cord arrangements deviating from the typical radial pattern (View 1), and significant hepatocyte swelling (View 2). In *Hacd2LKO* livers, hepatocytes showed marked nuclear abnormalities, including anisokaryosis (variably sized nuclei) and pyknosis (condensed nuclei), which are indicative of apoptotic processes. Notably, fragmented or absent nuclei were observed in partial hepatocytes alongside increased binucleation compared to WT livers with uniform nuclear morphology (View 3). Furthermore, a greater number of hepatocytes in *Hacd2LKO* mice exhibited ballooning degeneration and accumulation of lipid droplets (View 1–3). These features suggest that *Hacd2* deficiency may lead to the excessive production of triglycerides that cannot be adequately mobilized into the bloodstream or stored in adipose tissue, thereby contributing to hepatic lipid overload.

### 3.5. Hacd2 Deficiency Causes Liver Damage with Inflammation Under an LFHCD

**Figure 5:**
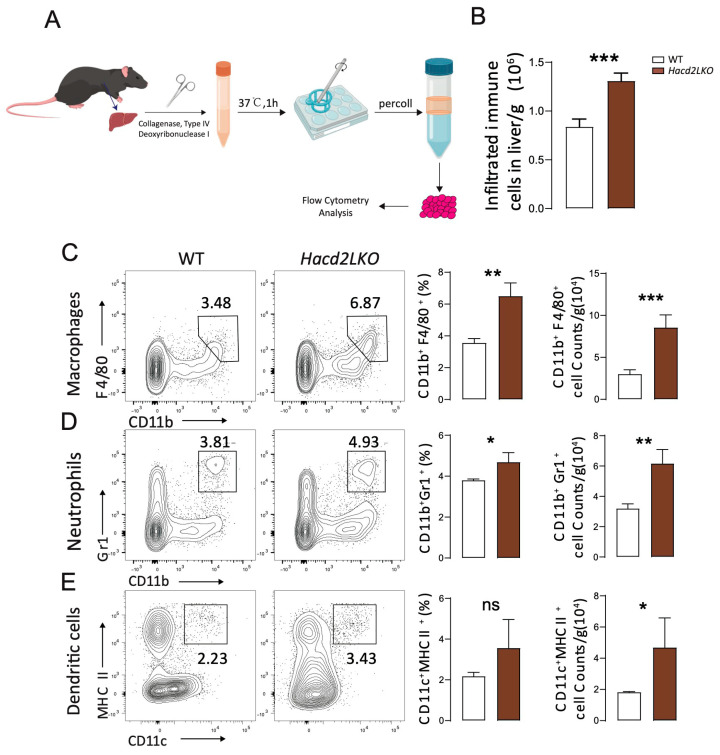
*Hacd2* deficiency causes liver damage with inflammation under an LFHCD. (**A**) Diagram of the process for the extraction of infiltrating immune cells from the liver [12]. (**B**) The number of infiltrated immune cells in the liver (WT n = 3, *Hacd2LKO* n = 5). (**C**–**E**) The proportions and absolute numbers of macrophages (**C**), neutrophils (**D**) and dendritic cells (**E**) infiltrating the liver (WT n = 3, *Hacd2LKO* n = 5). Data are presented as mean ± SEM, and statistical significance: *, *p* < 0.05; **, *p* < 0.01; ***, *p* < 0.001. ns, no significance.

*Hacd2* deficiency causes liver dysfunction, and abnormal hepatocytes may contribute to inflammation. H&E staining indicated a higher level of immune cells in the liver portal area or surrounding parenchyma (View 4, Figure 4K). To identify immune cell infiltration in the liver following *Hacd2* deficiency-induced injury, we enriched hepatic immune cells using Percoll density gradient centrifugation, which was followed by flow cytometric analysis (Figure 5A). The results demonstrated a significant increase in CD45^+^ immune cell infiltration in *Hacd2*-deficient livers (Figure 5B). Further immune subset analysis revealed marked expansion in the number of macrophages and neutrophils (Figure 5C,D), mediating both the phagocytic clearance of damaged hepatocytes and cytokine production for the promotion of inflammatory responses. Notably, dendritic cell numbers were also elevated, which exacerbated intrahepatic inflammation via enhanced antigen presentation ability (Figure 5E). Taken together, these data indicate that *Hacd2* deficiency impairs metabolic homeostasis, leading to decreased body weight and adipose tissue, and causes liver damage (as evidenced by increased dysfunction markers, abnormal hepatocytes and inflammation).

### 3.6. Hacd2 Is Essential for Triglyceride Synthesis During Adipocyte Differentiation

**Figure 6:**
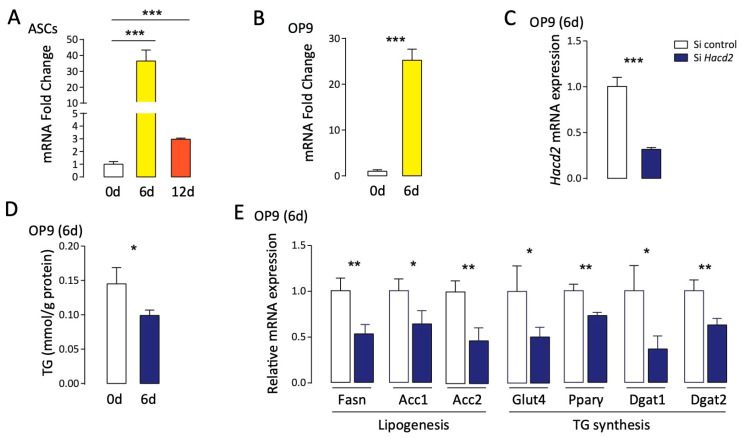
Increased *Hacd2* expression regulates triglyceride synthesis during adipocyte differentiation. (**A**) *Hacd2* mRNA expression during adipogenic differentiation of adipose-derived stem cells (ASCs). (**B**) *Hacd2* mRNA expression during adipocyte differentiation of OP9 cells. (**C**) *Hacd2* mRNA levels in control versus *Hacd2*-knockdown (*Hacd2*-KD) OP9 cells at day 6 of differentiation. (**D**) Triglyceride (TG) accumulation in control and *Hacd2*-KD OP9 cells at day 6 of differentiation. (**E**) Expression of adipogenic markers and TG synthesis-related genes in control and *Hacd2*-KD OP9 cells at day 6 of differentiation. Data are presented as mean ± SEM, and statistical significance: *, *p* < 0.05; **, *p* < 0.01; ***, *p* < 0.001.

Considering the roles of Hacd2 in maintaining metabolic homeostasis and liver function, we next addressed the function of Hacd2 in adipocytes. During the differentiation of adipose-derived stem cells (ASCs) and OP9 adipocytes, *Hacd2* mRNA expression was significantly increased. By day 6 of ASC differentiation, *Hacd2* transcript levels had risen more than 30-fold compared to those in undifferentiated cells, followed by a gradual decline as differentiation progressed (Figure 6A,B). To investigate the functional roles of Hacd2, we designed a small interfering RNA (siRNA) targeting a 654-bp sequence, achieving 70–80% knockdown efficiency (Figure 6C). On day 6 of OP9 adipocyte differentiation, the intracellular triglyceride (TG) content was significantly reduced in *Hacd2*-knockdown cells compared to controls (Figure 6D). Furthermore, the mRNA expression of key adipogenic genes—including Fasn (fatty acid synthase), ACC1/ACC2 (acetyl-CoA carboxylase), Glut4 (glucose transporter type 4), Ppar-γ (peroxisome proliferator-activated receptor gamma) and Dgat1/Dgat2 (diacylglycerol acyltransferase 1/2)—was markedly downregulated (Figure 6E). These results demonstrate that *Hacd2* deficiency disrupts both de novo lipogenesis and triglyceride synthesis pathways, highlighting its critical roles in adipocyte lipid metabolism.

## 4. Discussion

Fatty acid (FA) elongation is a four-step catalytic cycle involving condensation by Elovls, reduction by Hsd17b12, dehydration by Hacd2, and a final reduction by Tecr. Disruption of this cycle by impairing Elovl1 in the first step [13] or Hsd17b12 in the second step [14,15] has been shown to lead to defective embryonic development. In this study, we demonstrate that Hacd2 is essential for the synthesis of long-chain fatty acids (LCFAs) and that its deficiency causes embryonic lethality. Evidence suggests that LCFAs are stored as triglycerides in the yolk sac, serving as the primary energy source during embryogenesis [16]; for instance, LCFAs regulate embryonic development through the activation of nuclear receptors such as PPARα and NHR-49/80. In C. elegans, free palmitic acid activates NHR-49/80, promoting intestinal peroxisome migration and hormone secretion to drive late-stage embryogenesis [13]. Through single-cell sequencing, it has further been demonstrated that maternal hsd17b12a modulates intestinal LC-PUFA synthesis to affect yolk lipid absorption and endodermal organ development in the fetus [17]. Collectively, these findings indicate that Hacd2 could act as a potential checkpoint of embryonic development. Consistent with our findings, another study has reported that partial *Hacd2* knockout resulted in postnatal death with growth arrest, scurvy-like symptoms, and lethargy, whereas complete knockout caused more severe phenotypes, including developmental arrest and fatal cardiovascular malformations at E9.5. Furthermore, given the high expression of *Hacd2* in the testes, adipose tissue, and spleen, future research should explore the broader biological functions of Hacd2 beyond embryogenesis.

Embryonic lethality and liver dysfunction and damage indicate the indispensable roles of Hacd2 in maintaining organ development and metabolic homeostasis. However, the molecular pathways downstream of Hacd2 in embryonic and hepatic tissues remain poorly elucidated. A previous study has revealed that *Hacd2* deficiency triggers mitochondrial dysfunction, ultrastructural abnormalities, and oxidized lipoprotein accumulation, which contribute to embryo phenotype [18]. Hacd2 acts as a rate-limiting ER enzyme in VLCFA elongation, and its deficiency may affect membrane lipid composition, lipid-mediated signaling and organelle homeostasis. These disruptions could lead to cellular stress responses such as ER stress or mitochondrial dysfunction [19,20], which may underlie the developmental and metabolic phenotypes observed. For example, the ER is crucial for maintaining cell homeostasis as it is the primary site for the synthesis of secreted and transmembrane proteins and lipids. The interaction of VLCFA and PASTICCINO2 with Golgi anti-apoptotic proteins has been shown to confer resistance to endoplasmic reticulum stress [21]. Furthermore, Hacd2-mediated VLCFAs may be involved in other cellular functions, signal transduction in inflammatory responses and blood pressure regulation [22,23]; for example, they may regulate the lipoxygenase (LOX) and cyclooxygenase (COX) pathways, which are targeted by aspirin [24], activate macrophages through the TLR4/NF-κB pathway [25] or act as ligands interacting with PPARγ. Although these pathways remain unvalidated in our study, they provide a compelling rationale for future investigations [26].

Long-term abuse of a high-carbohydrate diet is as harmful as a high-fat diet for the development and progression of liver injury, as shown in a MAFLD mouse model [27]. Our current and previous studies systematically evaluated the dual roles of Hacd2 and revealed that its deficiency protects mice against HFD-induced obesity and MAFLD but impairs metabolic homeostasis under an LFHCD. Hacd2 can play a protective or harmful role under different metabolic dietary conditions, indicating that different nutrients in food alter hepatic metabolic pathways after ingestion. Under HFD conditions, the supply of exogenous fatty acids may partially compensate for the impaired synthesis of VLCFAs due to Hacd2 deficiency, thereby alleviating metabolic stress [28]. In contrast, LFHCD conditions increase the dependence on endogenous lipogenesis, rendering hepatocytes more susceptible to disruptions in fatty acid elongation. This susceptibility leads to lipid imbalances, ER stress, and compromised hepatocellular functions [29]. Moreover, high carbohydrate intake may activate lipogenic signaling pathways, further exacerbating hepatic lipid accumulation. Previous reports have indicated that, while LFHCD consumption does not cause significant weight gain, it induces more severe hepatic cholesterol deposition than high-fat, high-calorie diets in mice, primarily through the ASGR1-mediated mTORC1–USP20–HMGCR axis, which upregulates endogenous cholesterol synthesis [30]. Inhibition of ASGR1 upregulates LXRα, ABCA1 and ABCG5/G8 while inhibiting SREBP1 and lipogenesis, therefore promoting cholesterol excretion and decreased lipid levels [31]. While these mechanisms remain to be fully validated, we believe that they provide a plausible framework for understanding the diet-dependent hepatic outcomes observed in our study.

In conclusion, our study elucidated the critical role of Hacd2 in embryonic development and highlighted its dual function in alleviating metabolic burden and protecting against hepatic injury under an LFHCD. These findings thereby provide novel mechanistic insights for the therapeutic targeting of Hacd2 in metabolic diseases. However, several limitations remain, necessitating further investigation: (1) Whether the embryonic lethality induced by *Hacd2* deficiency involves non-enzymatic molecular mechanisms. (2) The divergent hepatic outcomes observed when targeting Hacd2, with its protective role under a high-fat diet versus its detrimental effects under an LFHCD, highlight that the regulatory mechanisms of Hacd2 remain poorly understood. (3) How Hacd2 regulates the downstream signaling pathways to present its metabolic effects remains to be comprehensively characterized, which may help to fully harness its therapeutic potential.

## 5. Conclusions

Our study demonstrated that Hacd2, a key enzyme in fatty acid elongation, is essential for embryonic development. Furthermore, through liver-specific *Hacd2* ablation (*Hacd2LKO*), we revealed that Hacd2 exerts dual functions in alleviating metabolic burden and protecting against hepatic injury under an LFHCD. These findings suggest that Hacd2 may serve as a therapeutic target for metabolic diseases. However, targeted intervention strategies must account for dietary variations and hepatic function.

## Figures and Tables

**Table 1:** Distribution of progeny genotypes of mice with different genotypes after mating.

Mating Genotype	Progeny Genotypes		
	*Hacd2* ^+/+^	*Hacd2* ^+/−^	*Hacd2* ^−/−^
♂ WT♀*Hacd2*^+/−^	68	72	0
♂ *Hacd2*^+/−^♀WT	68	66	0
♂ *Hacd2*^+/−^♀*Hacd2*^+/−^	169	272	0

## Data Availability

All data of the study are available on reasonable requirements from the corresponding author.
